# Spatiotemporal Regulation of Nuclear Transport Machinery and Microtubule Organization

**DOI:** 10.3390/cells4030406

**Published:** 2015-08-21

**Authors:** Naoyuki Okada, Masamitsu Sato

**Affiliations:** Laboratory of Cytoskeletal Logistics, Department of Life Science and Medical Bioscience, Graduate School of Advanced Science and Engineering, Waseda University, Center for Advanced Biomedical Sciences (TWIns), 2-2 Wakamatsucho, Shinjuku, Tokyo 162-8480, Japan; E-Mail: n.okada@aoni.waseda.jp

**Keywords:** cell cycle, fission yeast, nucleocytoplasmic shuttling, Ran-GTP, spindle, transport

## Abstract

Spindle microtubules capture and segregate chromosomes and, therefore, their assembly is an essential event in mitosis. To carry out their mission, many key players for microtubule formation need to be strictly orchestrated. Particularly, proteins that assemble the spindle need to be translocated at appropriate sites during mitosis. A small GTPase (hydrolase enzyme of guanosine triphosphate), Ran, controls this translocation. Ran plays many roles in many cellular events: nucleocytoplasmic shuttling through the nuclear envelope, assembly of the mitotic spindle, and reorganization of the nuclear envelope at the mitotic exit. Although these events are seemingly distinct, recent studies demonstrate that the mechanisms underlying these phenomena are substantially the same as explained by molecular interplay of the master regulator Ran, the transport factor importin, and its cargo proteins. Our review focuses on how the transport machinery regulates mitotic progression of cells. We summarize translocation mechanisms governed by Ran and its regulatory proteins, and particularly focus on Ran-GTP targets in fission yeast that promote spindle formation. We also discuss the coordination of the spatial and temporal regulation of proteins from the viewpoint of transport machinery. We propose that the transport machinery is an essential key that couples the spatial and temporal events in cells.

## 1. Introduction

Cells undergo spatiotemporal-specific phenomena through the cell cycle. The reorganization of microtubules is a good example of such a phenomenon [1,2,3,4]. During interphase, a cytoplasmic array of microtubules, which is drastically reorganized into spindle microtubules during mitosis, is assembled [5,6]. This reorganization is essential for chromosome segregation, because spindle microtubules emanating from two poles capture and segregate chromosomes. A number of proteins are known to be involved in the reorganization and, particularly, the translocation of those proteins to the right places with the right timing is a critical step to perform cell cycle-specific microtubule reorganization. A key question regarding the process is how cells can couple the time and the space.

Ran orchestrates the nucleocytoplasmic transport during interphase, but it plays a role in spindle formation during mitosis. Therefore, it appears that Ran is the critical factor that answers this question. Our review starts from the function of Ran as an organizer of transport machinery.

## 2. Research History of Ran-dependent Transport Machinery and Spindle Microtubule Assembly

### 2.1. Ran-Dependent Nucleocytoplamic Transport Machinery

A cell is divided into two areas: the nucleus and the cytoplasm. The nuclear envelope serves as a spatial barrier to compartmentalize the nucleus from the rest of the cell. Various biomacromolecules including proteins and RNAs need to shuttle between the nucleus and the cytoplasm, across the nuclear envelope [7]. In the last two decades, a huge amount of research determined that Ran conducts the nucleocytoplasmic shuttling through the transport machinery, including importin and exportin [8,9,10,11,12].

Ran is a small GTPase (hydrolase enzyme of guanosine triphosphate) that belongs to the Ras superfamily, originally identified in a human teratocarcinoma cell line [13]. Ran binds guanine nucleotides (GTP and GDP), and GTP-bound Ran exists predominantly in the nucleus. This is because the guanine nucleotide exchanging factor (GEF) for Ran, RanGEF (also called RCC1), is bound to the chromatin packaged in the nucleus [14,15,16].

In contrast, the GTPase activating protein (GAP) for Ran, RanGAP, localizes to the cytoplasm, which converts Ran-GTP to -GDP in the cytoplasm with its binding partner RanBP [17,18]. As a result of differential localization of RanGEF and RanGAP, Ran is spatially separated from the GTP-bound form and the GDP-bound form in the nucleus and the cytoplasm, respectively.

The contribution of Ran to nuclear transport was first shown in digitonin-permeabilized cells treated with *Xenopus laevis* cytosol extracts [19,20]. The molecular mechanism of how Ran orchestrates the transport was revealed step by step and, consequently, it was clarified that Ran modulates the interaction between two import factors: importin-α and importin-β [21].

**Figure 1:**
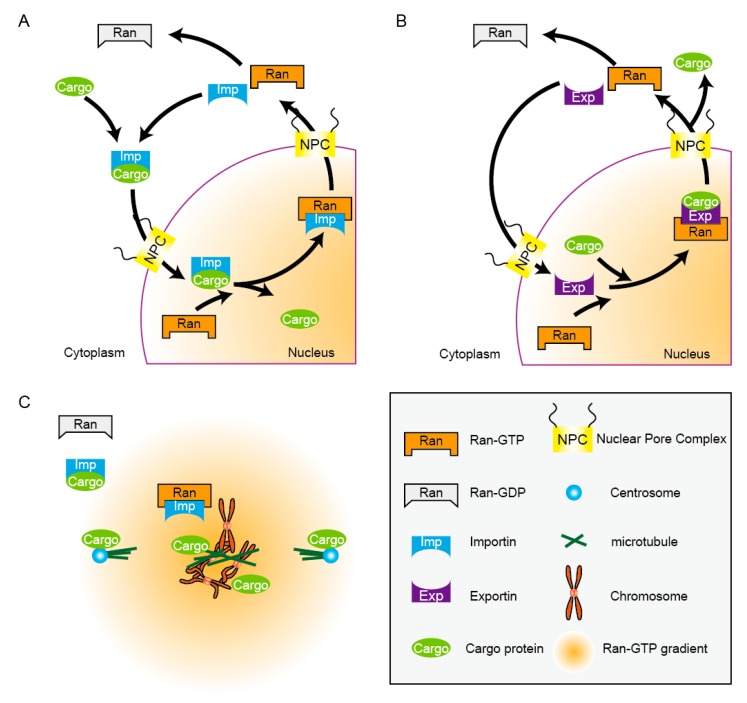
The nuclear transport cycle and spindle microtubule assembly governed by Ran-GTP. (**A**) A schematic of the nuclear import cycle. The importin-cargo complex assembles in the presence of Ran-GDP in the cytoplasm and its interaction with Ran-GTP unloads the cargo in the nucleus. (**B**) A schematic of the nuclear export cycle. Exportin and a cargo associate in the nucleus in the presence of Ran-GTP but dissociate in the cytoplasm. (**C**) In mitosis, the nuclear envelope breaks down (NEBD) and spindle microtubules are assembled in the close vicinity of chromosomes. Ran-GTP molecular gradient centered around the chromosomes dissociates the target molecules (cargos) through an interaction with importin.

Importin carries cargo molecules from the cytoplasm and unloads cargo in the nucleus, where Ran-GTP is enriched. The mechanism as to how a cargo protein is transported into the nucleus is as follows: importin-α functions as an adapter which recognizes the nuclear localization signal (NLS) of a cargo protein, and importin-β functions as a receptor molecule of the importin-α-cargo complex [22]. Importin-β transports the complex into the nucleus through the nuclear pore, which is channeled through the nuclear envelope and is surrounded by the gate—the nuclear pore complex (NPC) [7,23,24,25]. The importin-α-importin-β-cargo complex meets Ran-GTP in the nucleus, and causes dissociation of importin-α and the cargo [26,27]. This is the unloading step for the cargo (Figure 1A). In summary, the nuclear import of a cargo substantially comprises two major procedures: passing through the NPC and unloading in the nucleus. This is how cargo is accumulated in the nucleus.

Conversely, in the nuclear export, exportin, which belongs to the importin-β family, forms a complex with the cargo protein, which has the nuclear export signal (NES), in the presence of Ran-GTP, namely in the nucleus (Figure 1B) [28]. Formation of this exportin-cargo-Ran-GTP complex triggers its nuclear export through the NPC, and then this ternary complex is dissociated in the cytoplasm through an association with RanBP, followed by GTP hydrolysis of Ran-GTP stimulated by RanGAP [29,30]. As the knowledge of the transport machinery accumulated massively over decades, another important role for Ran was then unveiled at the end of the 1990s. Ran was identified as a crucial factor of microtubule assembly in cell-free experiments. In the next section, we focus on Ran as a key factor to promote microtubule assembly. Examples of the export cargo are shown in Table 1.

**Table 1:** Example of cargo exported from the nucleus to the cytoplasm.

Cargo	Class of Protein	Nuclear Export Receptor	Organism	Reference
Alp14	MAP	Crm1/Exportin-1	*S*. *pombe*	[31]
Klp5/Klp6	kinesin	Crm1/Exportin-1	*S*. *pombe*	[32]
Cyclin B1	B-type cyclin	Crm1/Exportin-1	*H*. *sapiens*	[33]
Survivin	CPC (Chromosome Passenger Complex) component	Crm1/Exportin-1	*M*. *musculus H*. *sapiens*	[34,35]
TPX2	MAP	Crm1/Exportin-1	*A*. *thaliana H*. *sapiens*	[36,37]
BRCA1	DNA damage response protein	Crm1/Exportin-1	*H*. *sapiens*	[38]
Pho4	transcription factor	Msn5/Exportin-5	*S*. *cerevisiae*	[39]
Srp1	importin-α	Cse1/CAS	*S*. *cerevisiae*	[40]

### 2.2. Molecular Gradient of Ran-GTP Navigates Spindle Assembly

Acentrosomal microtubule assembly was already suggested 30 years ago. Microtubule assembly guided by the chromatin was originally found through an experiment in which an injection of lambda DNA into *Xenopus laevis* eggs arrested in metaphase [41]. There was no clear answer as to how the chromatin guided microtubules at that time. Fifteen years after the discovery, several groups discovered that Ran-GTP is involved in microtubule assembly using *Xenopus* egg extracts [42,43,44,45,46]. For example, Karsenti and colleagues elegantly showed that GTP-bound Ran is crucial for microtubule assembly in the vicinity of chromosomes, using purified Ran and its mutant proteins, RCC1/RanGEF, and chromatin beads with mitotic extracts of *Xenopus* eggs [42,47]. Because the nuclear envelope breaks down during mitosis in higher eukaryotes, they proposed the hypothesis that the concentration of Ran-GTP in mitotic cells is maximized around chromosomes and is gradually decreased outwards and minimized in the periphery of the cell membrane.

These reports were the dawn of the biology of Ran-GTP in microtubule assembly, along with its canonical function in nuclear transport. The existence of the Ran-GTP molecular gradient was later verified through visualization of the gradient both in *Xenopus* egg extracts and human HeLa cells by the fluorescence resonance energy transfer (FRET) analyses [48,49,50]. Kalab *et al*. used two types of sensors for FRET in *Xenopus* egg extracts. One was RanBD (Ran-GTP binding domain of RanBP) fused with both YFP (Yellow fluorescent protein) and CFP (Cyan fluorescent protein) as YFP–RanBD–CFP, which undergoes FRET in the free state, but decreases FRET through a conformation change when bound to Ran-GTP. The second sensor was IBB (importin-beta binding: importin-β binding domain of importin-α) fused with YFP and CFP (YFP-IBB-CFP). In the absence of Ran-GTP, importin-β binds to YFP-IBB-CFP, thereby turning its FRET off. In contrast, in the presence of Ran-GTP, it sequesters importin-β, and the IBB of the sensor protein becomes free, which induces a conformational change to turn its FRET on. This system thus monitors the dissociation of importin-β/α and its cargo molecule, thereby visualizing a Ran-GTP gradient. A number of target proteins of Ran-GTP were also determined in parallel, which turned to be microtubule-associated proteins (MAPs), including TPX2, NuMA, and HURP [51,52,53,54,55,56]. Some of those targets contain NLS so that they are captured by the importin-β/α complex. In the close vicinity of chromosomes, Ran-GTP is abundant and it sequesters importin-β, thereby releasing these target proteins. Because the target proteins promote microtubule assembly, they are named spindle assembly factors (SAFs) (Figure 1C, Table 2).

Although these breakthroughs were made with *Xenopus* egg extracts and human cells, the Ran-GTP pathway for microtubule assembly is conserved in other organisms. For instance, siRNA-based studies demonstrated that the Ran pathway is critical for the assembly of spindle microtubules in *Caenorhabditis elegans* embryos [57]. It has also been shown that the injection of RanT24N (the GDP-locked mutant of Ran) to *Drosophila melanogaster* embryos induces perturbation of spindle assembly [58]. These reports indicate that Ran-GTP-dependent microtubule assembly is conserved in many organisms.

Ran thus plays two functions: one is nucleocytoplasmic shuttling during interphase, and the other is microtubule assembly during mitosis. It seems that these two phenomena are completely distinct in time and in purpose, but the underlying mechanisms are basically similar. It is notable that these two mechanisms are based on capture and release of “cargo” proteins by importin-β/α and the balance between Ran-GTP and Ran-GDP. The only difference is whether the nuclear envelope serves as a barrier for the Ran-GTP gradient. GTP-bound Ran functions as the spatial cue that signals the destination of cargos, *i.e*., cargos are released where Ran-GTP exists. In the interphase, Ran-GTP is enriched inside the nuclear envelope where imported cargos with NLS are unloaded. The cargo proteins for microtubule assembly are, in general, MAPs, which are released in the vicinity of chromosomes [55,59] where the Ran-GTP concentration is highest, although the nuclear envelope is broken down.

**Table 2:** List of microtubule-associated proteins targeted by Ran GTPase.

Cargo	Class of Protein	Nuclear Import Receptor	Organism	Reference
TPX2	MAP	Importin-α/β	*X*. *laevis*	[51]
NuMA	MAP	Importin-α/β	*X*. *laevis*	[53,54]
HURP	MAP	Importin-β	*H*. *sapiens*	[55,56]
NuSAP	MAP	Importin-α/β/Importin-7	*X*. *laevis, H*. *sapiens*	[60]
Alp7 (TACC)	MAP	Cut15/Importin-α	*S*. *pombe*	[61]
Maskin (TACC)	MAP	Importin-β	*X*. *laevis*	[62]
MCRS1	MAP	Importin-β	*X*. *laevis*	[63]
Xnf7	MAP	Importin-β	*X*. *laevis*	[64]
Kid	Chromokinesin	Importin-α/β	*H*. *sapiens*	[36,65]
XCTK2	Kinesin-14	Importin-α/β	*X*. *laevis*	[66]

Thus, Ran-GTP is a master regulator of the spatial distribution of proteins in cells. To achieve a number of cellular events with the right timing, Ran-GTP needs to release specific cargos in the right place at the right time. For instance, to assemble the spindle, Ran’s targets need to function in the close vicinity of chromosomes during mitosis. The release of Ran’s targets must be coordinated with the progression of the cell cycle. How can these two events be coupled? Our group found out a clue for the answer through our recent studies regarding Ran’s target in fission yeast.

## 3. Ran-GTP Promotes Formation of Spindle Microtubules in Fission Yeast

In many single-celled eukaryotic organisms such as fission yeast, the nuclear envelope remains intact during mitosis and, consequently, chromosomes are segregated inside the nucleus [67]. This type of mitosis is called “closed mitosis”, in contrast to the “open mitosis” of higher eukaryotes, in which the nuclear envelope breaks down at the mitotic entry.

In closed mitosis, the spindle is organized in the compartmentalized nucleus, therefore factors required for spindle formation must be imported to the nucleus. This suggests that the Ran-GTP-dependent nuclear transport system operates even during mitosis, and that Ran-GTP regulates a number of targets to assemble the spindle. How does Ran-GTP control spindle assembly during closed mitosis? Is the nuclear transport regulated temporally to achieve Ran-dependent microtubule formation only during mitosis? To address these questions, we chose the fission yeast *Schizosaccharomyces pombe* as a model system and investigated how the spindle organization and the nuclear transport are coordinated.

**Figure 2:**
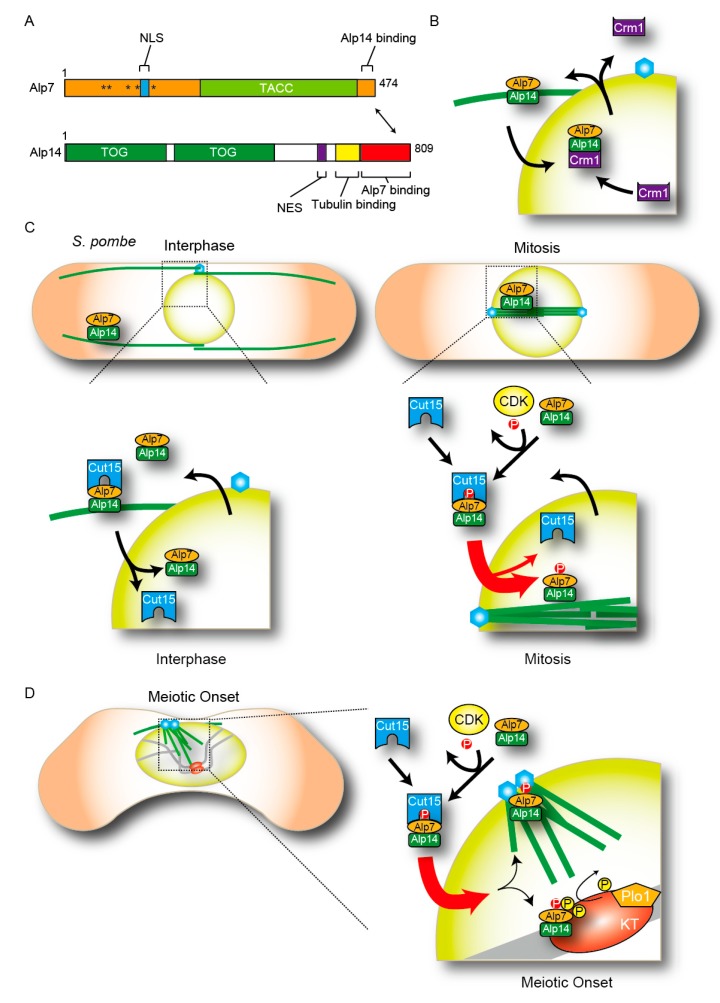
Nucleocytoplasmic shuttling of the Alp7-Alp14 complex in fission yeast. (**A**) Architectures of Alp7/TACC and Alp14/TOG. Alp7 has a nuclear localization signal (NLS) in its N-terminal half, whereas Alp7 interacts with Alp14 through its C-terminal end. Alp14 has a nuclear export signal (NES) in its middle region. Asterisks show the cyclin-dependent kinase (CDK) phosphorylation sites in Alp7. (**B**) The export of the Alp7-Alp14 complex. The NES in Alp14 plays a role in the nuclear export of the complex. (**C**) How the nuclear import of Alp7-Alp14 is modulated through the cell cycle. Left: During interphase, Alp7-Alp14 shuttles between the nucleus and the cytoplasm through the association of the NLS in Alp7 and importin-α/Cut15. Right: During mitosis, CDK phosphorylates Alp7 to accelerate the import activity of the complex and to promote spindle assembly. (**D**) Localization of Alp7-Alp14 at the onset of meiosis I. Alp7-Alp14 localizes to kinetochores (KT) even before microtubules that emanated from spindle pole bodies (SPBs) attach to the kinetochores.

Targets of Ran had not been identified in yeast at that time, and we thus started to identify them in fission yeast. We particularly focused on the Alp7/TACC protein (also called Mia1) as a SAF in *S*. *pombe*. Alp7 is an ortholog of the conserved TACC (transforming acidic coiled-coil) protein [68]. Alp7 has the conserved TACC domain at the C-terminus, through which it interacts with Alp14/TOG, an ortholog of a conserved MAP, ch-TOG/XMAP215 [69,70] (Figure 2A). The MAP complex tracks the plus-end of a microtubule bundle and regulates the dynamics [71,72]. The Alp7-Alp14 complex localizes to cytoplasmic microtubules in interphase. As cells enter mitosis, Alp7-Alp14 accumulates in the nucleus and localizes to spindle microtubules, spindle pole bodies (SPBs, the centrosome equivalent in yeast), and the kinetochore periphery [70,73,74,75,76,77]. Importantly, Alp7 contains an NLS in the N-terminal (non-TACC) domain. The *alp7-RARA* mutant, which has mutations in the NLS, shows severe defects including the monopolar spindle phenotype, indicating that nuclear accumulation of Alp7-Alp14 during mitosis is crucial for spindle assembly [61].

We thus identified Alp7/TACC-Alp14/TOG as a crucial target of Ran in fission yeast [61]. Alp7-Alp14 accumulates in the nucleus during mitosis to assemble the spindle, but it does not during interphase. This time-specific behavior of the MAP complex led us to consider that the mechanism of its nuclear transport system may give us some clues for the questions as to how temporal and spatial regulations of spindle assembly are coordinated, and how the nuclear transport of cargo proteins can be altered depending on the cell cycle stages.

To address those questions, we need to assemble the overall picture describing how the Alp7-Alp14 complex is transported, particularly from the viewpoint of nuclear export. The MAP complex loses nuclear accumulation upon mitotic exit towards interphase; therefore, the complex is expected to harbor the nuclear export signal (NES). We set out to construct a series of truncation mutants of Alp14 and mapped a region essential for the Alp14 export in the middle region (Figure 2B) [31]. A reporter protein containing the corresponding region fused with GST-GFP stayed in the cytoplasm constitutively. This experiment verified that the region functions as an NES, which is recognized by exportin/Crm1. The NES of Alp14 contains a leucine-rich sequence and a single mutation of a leucine residue caused a loss of the NES activity. The nuclear export of the Alp7-Alp14 complex is indeed dependent upon the NES of Alp14, and we concluded that Alp7 and Alp14 behave as a complex both at nuclear import and export.

The Alp7-Alp14 complex enters the nucleus using the NLS of Alp7, whereas it exits using the NES of Alp14. We found that the Alp7-Alp14 complex shuttles between the nucleus and the cytoplasm throughout the cell cycle. Leptomycin B, an inhibitor of exportin/Crm1 [78], causes nuclear accumulation of Alp7-Alp14 even in interphase cells [75]. These results demonstrate that the complex normally enters the nucleus even during interphase, but is excluded from the cytoplasm; therefore, the complex predominantly localizes to cytoplasmic microtubules and its nuclear localization is not evident in interphase cells.

Having described the overall picture as to how the Alp7-Alp14 complex behaves, we next figured out how the nuclear transport of the complex is regulated in a cell cycle-dependent manner. Our group showed that the nuclear accumulation of Alp7-Alp14 is under the control of the cyclin-dependent kinase Cdc2/CDK1 [75], although its molecular mechanism had been unknown. As the Alp7-Alp14 complex undergoes nucleocytoplasmic shuttling even during interphase, we suspected two major possibilities for the mitosis-specific nuclear accumulation as follows: (1) the nuclear import of Alp7-Alp14 is accelerated during mitosis; or (2) the nuclear export of the complex is inhibited during mitosis, although these two are not mutually exclusive. The first possibility indicates that the NLS region of Alp7 might be post-transcriptionally modified, such as phosphorylation by CDK, for instance. The latter implies that the NES region of Alp14 could be regulated by CDK. As nuclear accumulation of the Alp7-Alp14 complex does not seem to rely on sequestration by any nuclear architecture (such as the nuclear envelope or chromatin), we ruled out the “third” possibility that the association of the complex to a nuclear architecture is the key for mitosis-specific translocation.

To investigate how CDK influences Alp7-Alp14 localization, we searched for phosphorylation of the complex by CDK, and found that Alp7 is phosphorylated by CDK *in vitro* [31]. We then identified five phosphorylation sites nearby the NLS in the N-terminus through kinase assays using truncation and point mutants of Alp7 (Figure 2A). Remarkably, phosphorylation of these five sites by CDK enhances the interaction between the NLS of Alp7 and importin-α/Cut15 during mitosis (Figure 2C). This causes a mitosis-specific acceleration of nuclear import of Alp7-Alp14. CDK thus causes a temporal imbalance of direction of Alp7-Alp14 transport. In summary, CDK regulates localization of a crucial SAF of Ran-GTP during mitosis in fission yeast.

These findings gave us some interesting hypotheses about evolutionary conservation and divergence regarding Ran-mediated control of microtubule organization.

First, does Ran-GTP form a molecular gradient in fission yeast, as higher eukaryotes do? The Ran-mediated transport machinery is conserved from yeast to human. Persistence of the nuclear envelope during mitosis restricts the distribution of Ran’s targets in the closed mitosis organism: the cell size of fission yeast may be too small to compose the spatial gradient of Ran-GTP, while the nuclear envelope is the spatial barrier to limit the dispersion of Ran’s targets in this organism.

Second, targets of Ran GTPase for microtubule assembly appear divergent in species. Fission yeast utilizes Alp7/TACC as a crucial target, whereas higher eukaryotes utilize TPX2, NuMA, HURP, and so on. Interestingly, many of those factors do not have apparent orthologs in yeast. We speculate that those targets might be evolved to assemble larger and more complicated spindles of metazoa than that of yeast. TACC and XMAP215/TOG in metazoa have been identified as proteins that interact with Ran’s targets [55,62]. Therefore, they could be universal targets of Ran, required for spindle assembly from yeast to human.

Taking those speculations together, Alp7/TACC might have been chosen as an optimal target of Ran in fission yeast. Since the spatial barrier restricts formation of the Ran-GTP gradient, cells might need to assemble a compact spindle exclusively from SPBs. To achieve this, Alp7/TACC may be the best candidate because it localizes to both SPBs and microtubules and interacts with the microtubule polymerase Alp14/TOG.

In addition, Alp7/TACC plays a particular function at kinetochores in meiosis. It has been known that in mitosis, Alp7-Alp14 is brought to kinetochores by microtubules only when the microtubule plus-end attaches to kinetochores [73,76,77,79]. Alp7/TACC also localizes to kinetochores before entry into the first division of meiosis, but the situation is completely different from mitosis. In meiosis, Alp7/TACC localizes to kinetochores even before microtubule attachment [74] (Figure 2D). Kinetochores are scattered in the nucleus at the stage of meiosis [80,81,82], and the Polo-like kinase localizes to the scattered kinetochores and phosphorylates Alp7/TACC to recruit it there, even before microtubule attachment (Figure 2D). This precocious localization of Alp7/TACC observed specifically in meiosis is required for the scattered kinetochores to be captured by microtubules [74]. Ran might place its target Alp7/TACC to meiotic kinetochores in order to employ it for meiosis-specific functions in kinetochore capturing. It is tempting to investigate whether kinetochore-mediated microtubule nucleation occurs in fission yeast meiosis, although we have currently no visual evidence for it.

Thus, the pinpointed localization of Ran’s target could be sufficient to organize a small spindle in the nucleus of fission yeast. These findings gave us some interesting hypotheses regarding evolutionary divergence in the spatiotemporal control of proteins. In fission yeast, the size of the mitotic spindle is relatively smaller and the structure appears simpler than that of higher eukaryotes, hence only few proteins are sufficient to change their localization to the nucleus at mitotic entry compared to those in metazoa. Therefore, spatial and temporal control of microtubule assembly could be coordinated easily by regulating the transport of a small number of Ran’s targets. In higher eukaryotes, in order to construct a large spindle, the number of microtubule nucleation sites is increased, which might be a reason why a larger number of Ran’s targets must be required. Those cells might have adopted to break down the nuclear envelope rather than to import many targets into the mitotic nucleus, in order to coordinate microtubule assembly spatially and temporally. Both types of organisms successfully accomplish the spatiotemporal control of proteins without modulating the Ran system itself throughout the cell cycle.

## 4. Temporal Control of the Nucleocytoplasmic Shuttling

### 4.1. The Regulation of Temporal Nuclear Accumulation of Shuttling Proteins during the Cell Cycle

Our research thus revealed that in the case of Alp7/TACC, mitosis-specific nuclear accumulation of a transport cargo is achieved by acceleration of the nuclear import by CDK. How is temporal alteration of the nucleocytoplasmic localization achieved in other shuttling proteins, in other organisms?

There are mainly five categories for cell cycle-dependent alterations of the cargo localization; temporal modulation could be made by activation or inactivation of the export or import activity of the cargo, or by controlling transporters or components of the nuclear pore complex (nucleopolins; Nups).

**Figure 3:**
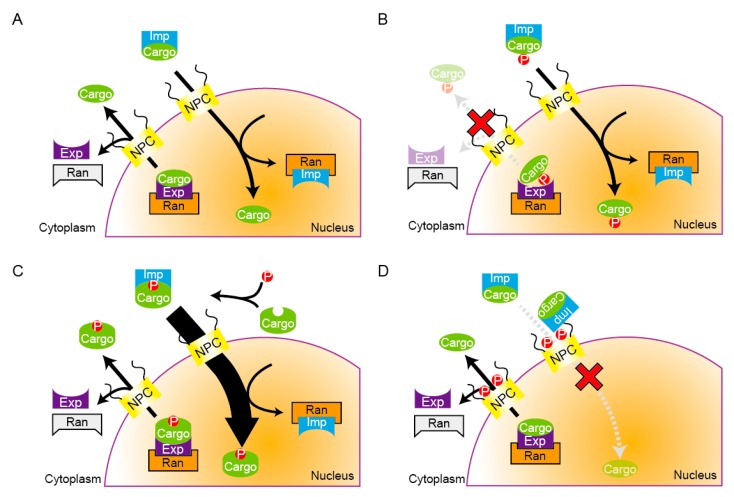
Patterns of temporal regulation of nuclear transport. (**A**) A schematic of the nuclear transport cycle of shuttling proteins. (**B**) An example of the regulation of the nuclear export. Temporal accumulation of cargo proteins can be achieved by shutting off the nuclear export through modification to the NES in cargos. (**C**) An example of the regulation of the nuclear import. Temporal accumulation of cargo here is due to acceleration of import through modification to the NLS of cargos; (**D**) An example of the regulation of nucleoporins (Nups). Phosphorylation of the nuclear pore complex (NPC) component blocks the nuclear import as importin is trapped at the NPC.

First, shutting off the nuclear export of a cargo would be largely achieved by the inactivation of the NES of the cargo (Figure 3A,B). For instance, Cyclin B1 contains an NES in its N-terminus, and shuttles continuously between the nucleus and the cytoplasm in HeLa cells. Its phosphorylation near the NES reduces its nuclear export activity, which consequently results in nuclear accumulation of Cyclin B1 [33].

Second and third, activation of the NES and inactivation of the NLS has been well studied in the regulation of Pho4 in budding yeast [83]. Pho4 is a transcription factor that activates the expression of genes induced in response to phosphate starvation [84]. Pho4 has three phosphorylation sites that contribute to its nucleocytoplasmic transport. These sites are phosphorylated by the Pho80/Pho85 cyclin-CDK complex, whose activity depends on phosphate conditions [85,86]. When cells are in phosphate starvation, unphosphorylated Pho4 accumulates in the nucleus. When phosphate is supplied, conversely, rapid phosphorylation occurs to Pho4 to export it from the nucleus. Phosphorylation at two of three sites promotes the association of Pho4 to Msn5, which is the export receptor belonging to the importin-β family [83]. Thus, the phosphorylation promotes the nuclear export through activation of the NES. Phosphorylation at the other site within the NLS inhibits its interaction to the import receptor Pse1, indicating that the phosphorylation blocks the nuclear import through inactivation of the NLS. Thus, to achieve temporal nuclear exclusion, Pho4 uses the hybrid pattern of two regulatory mechanics: activation of NES and inactivation of NLS.

Fourth, there are some proteins in which phosphorylation of nearby NLS enhances its activity (Figure 3C). As mentioned earlier, Alp7 is categorized here. Budding yeast Dna2 involved in the repair of DNA double-strand breaks accumulates to the nucleus from the DNA synthesis phase (S phase) to mitosis and resides in the cytoplasm during the G1 phase. The NLS activity of Dna2 turned out to be enhanced through phosphorylation by CDK [87]. Recently, the phosphorylation of NLS-adjacent sites by CDK was investigated in a human proteome analysis [88]. The authors found that the position of the phosphorylation site right before the NLS sequence matters. Mimicking the phosphorylation of a single residue ahead of the NLS causes a reduction in the NLS activity, whereas phospho-mimicking of a site two residues ahead of the NLS results in the activation of the NLS activity. NLS-adjacent phosphorylation may thus bring out distinct effects depending upon its position.

The last option is the regulation of transporters or Nups (Figure 3D). For example, in budding yeast, a nuclear pore complex protein, Nup53, changes its interaction with importin-β/Kap121 depending upon cell cycle stages [89,90]. In interphase, nucleoporins Nup53 and Nup170 interact with each other and locate inside the NPC. During mitosis, the phosphorylation of Nup53 by CDK dissociates Nup53 from Nup170, which unmasks Nup53’s interaction site for Kap121. Kap121 is then trapped to the NPC through the interaction with Nup53. As a result, the nuclear import via importin-β/Kap121 is repressed during mitosis. In another study using HeLa cells, Crm1/exportin is phosphorylated by CDK, which is required to form a complex with RanBP2 and RanGAP1 on spindle microtubules [91]. It is speculated that this complex enhances local GTP-hydrolysis of Ran-GTP on spindle microtubules, thereby promoting the dissociation of cargo proteins from Crm1 at the spindle.

When cells want to alter the nucleocytoplasmic localization of a certain kind of protein only transiently, regulating the cargo rather than regulating the transport system, *per se*, is the simplest way to achieve this. The simple way is likely to fit simple organisms such as yeast. On the other hand, the regulation of the system itself, e.g., transporters or Nups, may be useful in order to alter localization of various proteins at the same time. We suppose that modulating the transport system *per se*, rather than modulating each cargo, would be advantageous to globally regulate events in which multiple factors are involved. Even for simple organisms undergoing closed mitosis, such temporal regulation of transporters or Nups could imitate the situation as in the NEBD. As we mentioned above, the phosphorylation of Nup53 can block translocation of many kinds of cargo carried by Kap121 at the same time. The regulation of Crm1 in HeLa cells can also control the localization of a number of downstream targets.

In all cases, the temporal imbalance between import and export activities is one of the key events for the coordination of the cell cycle and protein localization. This coordination is essential to perform cell cycle-specific events in cells. As we discussed with examples, the most drastic and important player to arrange this spatiotemporal coordination is CDK. It is notable that many factors that play essential roles in cell cycle events are regulated at the level of protein localization, as well as at the level of enzymatic activity or protein interaction. Regulation of protein translocation might be the most direct way among various types of regulation in order to control protein localization in space and in time.

### 4.2. The Nuclear Envelope Permeability Changes during Mitosis in Several Organisms

Permeabilization of the nuclear envelope is another way to alter nucleocytoplasmic localization during the cell cycle. Even for organisms that undergo closed mitosis, it is known that the membrane permeability changes during M phase at some specific occasions. This phenomenon is often known as “semi-open” mitosis.

In early embryos of *C*. *elegans*, semi-open mitosis takes place: the permeability of the nuclear envelope is increased during mitosis. The accumulation of free tubulin in the pronuclear area is observed, which coincides with the permeabilization of the nuclear envelope during prometaphase [92]. The accumulation of free tubulin is critical for the subsequent assembly of a nascent spindle. RAN-1/Ran is involved in the tubulin accumulation, and it is intriguing to investigate the molecular mechanism of how Ran induces permeabilization in the nuclear envelope only in the specific stages of early development in *C*. *elegans*.

Semi-open mitosis is also observed in the filamentous fungus *A*. *nidulans*. In interphase, both tubulin dimers and RanGAP (called An-RanGAP) locate in the cytoplasm in the organism. During mitosis, however, both enter the nucleus [93]. This is caused by partial disassembly of the NPC, which is regulated by mitotic kinases NIMA and CDK. An NPC component, Nup98, disperses from the nuclear envelope during mitosis, which is impaired when NIMA or CDK is artificially inactivated.

In the fission yeast *S*. *pombe*, nuclear compartmentalization is abolished during the anaphase of meiosis II [94,95], but not in other cell cycle stages including meiosis I. In these studies, the authors found that the nuclear envelope permeability increases, and this is related to spore formation (yeast gametogenesis). In *S*. *pombe*, the forespore membrane is formed during meiosis II, which starts to assemble from the SPB located at the nuclear periphery, and encapsulates the nucleus as it grows (reviewed in [96]). The forespore membrane is generated through fusion of membrane vesicles generated at the ER (endoplasmic reticulum) via the Golgi apparatus. For the expansion of the nuclear envelope, membrane lipid is normally supplied from the ER [97,98] during the anaphase of mitosis or meiosis I. In contrast, in meiosis II, we speculate that membrane vesicles are used for forespore membrane assembly, which causes the temporary shortage of membrane components during anaphase II. As a result, it may cause an increase of nuclear envelope permeability. This was shown by blocking the ER-to-Golgi membrane vesicle transport using the drug Brefeldin A, which inverses the ER-to-Golgi pathway [94]. The GTPase-activating protein for Ran, RanGAP/Rna1, normally localizes to the cytoplasm, but it changes its localization from the cytoplasm to the nucleus during this phase. This would next invalidate the Ran-dependent transport machinery during anaphase II. In the fission yeast *Schizosaccharomyces japonicus*, which is one of the closest species to *S*. *pombe*, the nuclear envelope is physically torn in the mitotic anaphase, and the localization of nuclear pores on the nuclear envelope is biased in this phase [99]. It is speculated that the nuclear envelope is torn in the region which contains the lesser number of nuclear pores. The breakage of the nuclear envelope causes leakage of a nuclear protein, mCherry-NLS, suggesting that the dispersion occurs during anaphase. It is possible that the dispersion of proteins caused by the nuclear envelope breakage may assist stable elongation of the anaphase spindle in this organism.

It is not yet clearly defined whether closed or semi-open mitosis occurs in a social amoebozoan, *Dictyostelium discoideum* [100]. During mitosis, many kinds of proteins translocate by nucleocytoplasmic diffusion, indicating that this organism undergoes semi-open mitosis. Although tubulin dimers are excluded from the nucleus during interphase, they mainly accumulate in the nucleus during mitosis [101]. This implies the NPC-mediated nuclear import machinery operates despite semi-open mitosis.

Little is known regarding how the semi-open mitosis is conducted and how protein transport is regulated. As mentioned above, fission yeast appears to have two modes of nuclear divisions: closed mitosis and semi-open meiosis II. It would be interesting to investigate whether other organisms, which are known to undergo closed mitosis, also undergo semi-open mitosis only on special occasions. As semi-open mitosis is considered the intermediate version between “open” and “closed” mitoses, it would give rise to a moderate transport, which may be useful at some occasions that require only partial translocation of proteins. Further research in the near future will provide new insights about the evolution of the regulation system of nucleocytoplasmic transport machinery.

## 5. The Regulation of Translocation of MAPs and Spindle Microtubule Organization

MAPs need to be translocated at proper sites for the assembly of the bipolar spindle [6,102,103]. Cell cycle-dependent translocation of several MAPs has been well studied [104,105]. It seems that in most of the cases, CDK regulates the nucleocytoplasmic translocation of MAPs through phosphorylation.

In fission yeast, it is already known that Ase1/PRC1 is a microtubule bundler that helps spindle elongation during anaphase [106,107,108]. Ase1 is phosphorylated at several sites by CDK and the phosphorylation is required for the nuclear translocation of Ase1 during mitosis [109]. Another role of CDK phosphorylation in Ase1 is to inhibit its association to Klp9/kinesin-6 on the spindle in metaphase [109]. The phosphorylation by CDK prevents premature recruitment of Klp9 to the spindle midzone, thereby blocking premature elongation of spindle microtubules during metaphase.

In another case, a heterodimer of kinesin-8 family proteins in fission yeast, Klp5-Klp6, shuttles between the cytoplasm and the nucleus through the cell cycle [32,110]. It remains to be elucidated how the phosphorylation events lead to temporal changes of their localization, but it is known that CDK consensus sequences reside near their NLSs. It is possible that the NLS of the kinesin dimer is also activated during mitosis by CDK phosphorylation.

In HeLa cells, NuSAP (nuclear and spindle assembly protein) changes its localization in the metaphase-to-anaphase transition [111]. Localization of NuSAP to the spindle midzone is inhibited by CDK phosphorylation during metaphase, and its dephosphorylation in anaphase promotes NuSAP to bundle microtubules at the midzone.

A recent study shows that another MAP, TPX2, is phosphorylated at Thr72 by CDK1/2, which affects its spindle localization [112]. When the phosphorylation is abolished, TPX2 becomes abnormally accumulated in spindle microtubules and the multipolar spindle is frequently observed. The altered localization of the TPX2-T72A mutant protein causes enhancement of the activity of Aurora-A at the spindle pole.

## 6. Conclusions

The nuclear envelope is the essential shelter that isolates the genomic DNA from the rest of the organelles in eukaryotic cells. Although the compartmentalization brings precise and elaborate systems of cell regulation, it might also need complexity in regulation of the cell cycle progression: the spindle needs to be built in M phase, when the genome is divided into two nuclei for two daughter cells. Eukaryotic cells solve this issue by temporary alteration of the nucleocytoplasmic transport.

The ultimate way to change the state of nucleocytoplasmic shuttling is to break down the nuclear envelope. Although this is a rigid strategy that globally changes the localization of most compartmentalized proteins, it retains the Ran-dependent transport machinery and is appropriate for dynamic cellular events such as mitotic spindle assembly. Indeed, many higher organisms take this tactic for spindle assembly. Simpler eukaryotes in which the nuclear envelope persists during mitosis have adopted and evolved with simpler methods to temporally modify a part of the transport machinery. In these systems, transport cargo or transport factors receive post-transcriptional regulations to change their transport status only for themselves.

It is CDK that links the cell cycle- and Ran-dependent nuclear transport of proteins, as its activity is oscillated throughout the cell cycle. The temporal and spatial regulation of events in the cell are perfectly coordinated by two collaborative conductors, CDK and Ran.

## References

[1] Mitchison T., Evans L., Schulze E., Kirschner M. (1986). Sites of microtubule assembly and disassembly in the mitotic spindle. Cell.

[2] Kirschner M., Mitchison T. (1986). Beyond self-assembly: From microtubules to morphogenesis. Cell.

[3] Hayles J., Nurse P. (2001). Access: A journey into space. Nat. Rev. Mol. Cell Biol..

[4] Kiyosue Y.M. (2011). Shaping microtubules into diverse patterns: Molecular connections for setting up both ends. Cytoskeleton.

[5] Hagan I., Hyams J. (1988). The use of cell division cycle mutants to investigate the control of microtubule distribution in the fission yeast Schizosaccharomyces pombe. J. Cell Sci..

[6] Sato M., Toda T. (2010). Space shuttling in the cell: Nucleocytoplasmic transport and microtubule organization during the cell cycle. Nucleus.

[7] Izaurralde E., Adam S. (1998). Transport of macromolecules between the nucleus and the cytoplasm. RNA.

[8] Ohno M., Fornerod M., Mattaj I.W. (1998). Nucleocytoplasmic transport: The last 200 nanometers. Cell.

[9] Sazer S., Dasso M. (2000). The ran decathlon: Multiple roles of Ran. J. Cell Sci..

[10] Moore J.D. (2001). The Ran-GTPase and cell-cycle control. Bioessays.

[11] Dasso M. (2001). Running on Ran: Nuclear transport and the mitotic spindle. Cell.

[12] Clarke P.R., Zhang C. (2001). Ran GTPase: A master regulator of nuclear structure and function during the eukaryotic cell division cycle?. Trends Cell Biol..

[13] Drivas G., Shih A., Coutavas E., Rush M. (1990). Characterization of four novel ras-like genes expressed in a human teratocarcinoma cell line. Mol. Cell. Biol..

[14] Ohtsubo M., Okazaki H., Nishimoto T. (1989). The RCC1 protein, a regulator for the onset of chromosome condensation locates in the nucleus and binds to DNA. J. Cell Biol..

[15] Bischoff F.R., Ponstingl H. (1991). Catalysis of guanine nucleotide exchange on Ran by the mitotic regulator RCC1. Nature.

[16] Matsumoto T., Beach D. (1991). Premature initiation of mitosis in yeast lacking RCC1 or an interacting GTPase. Cell.

[17] Bischoff F., Krebber H., Smirnova E., Dong W. (1995). Co-activation of RanGTPase and inhibition of GTP dissociation by Ran-GTP binding protein RanBP1. EMBO J..

[18] Bischoff F., Klebe C., Kretschmer J. (1994). RanGAP1 induces GTPase activity of nuclear Ras-related Ran. Proc. Natl. Acad. Sci. USA.

[19] Melchior F., Paschal B., Evans J., Gerace L. (1993). Inhibition of nuclear protein import by nonhydrolyzable analogues of GTP and identification of the small GTPase Ran/TC4 as an essential transport factor. J. Cell Biol..

[20] Moore M.S., Blobel G. (1993). The GTP-binding protein Ran/TC4 is required for protein import into the nucleus. Nature.

[21] Rexach M., Blobel G. (1995). Protein import into nuclei: Association and dissociation reactions involving transport substrate, transport factors, and nucleoporins. Cell.

[22] Görlich D. (1997). Nuclear protein import. Curr. Opin. Cell Biol..

[23] Ström A.C., Weis K. (2001). Importin-ß-like nuclear transport receptors. Genome Biol.

[24] Fried H., Kutay U. (2003). Nucleocytoplasmic transport: Taking an inventory. Cell. Mol. Life Sci..

[25] Wente S.R., Rout M.P. (2010). The Nuclear Pore Complex and Nuclear Transport. Cold Spring Harb. Perspect. Biol..

[26] Chi N., Adam E., Visser G. (1996). RanBP1 stabilizes the interaction of Ran with p97 nuclear protein import. J. Cell Biol..

[27] Görlich D., Pante N., Kutay U., Aebi U. (1996). Identification of different roles for RanGDP and RanGTP in nuclear protein import. EMBO J..

[28] Fornerod M., Ohno M., Yoshida M., Mattaj I. (1997). CRM1 is an export receptor for leucine-rich nuclear export signals. Cell.

[29] Bischoff F.R., Görlich D. (1997). RanBP1 is crucial for the release of RanGTP from importin β-related nuclear transport factors. FEBS Lett..

[30] Koyama M., Matsuura Y. (2010). An allosteric mechanism to displace nuclear export cargo from CRM1 and RanGTP by RanBP1. EMBO J..

[31] Okada N., Toda T., Yamamoto M. (2014). CDK-dependent phosphorylation of Alp7-Alp14 (TACC-TOG) promotes its nuclear accumulation and spindle microtubule assembly. Mol. Biol. Cell.

[32] Unsworth A., Masuda H., Dhut S., Toda T. (2008). Fission yeast kinesin-8 Klp5 and Klp6 are interdependent for mitotic nuclear retention and required for proper microtubule dynamics. Mol. Biol. Cell.

[33] Yang J., Bardes E., Moore J., Brennan J. (1998). Control of cyclin B1 localization through regulated binding of the nuclear export factor CRM1. Genes Dev..

[34] Knauer S.K., Bier C., Habtemichael N., Stauber R.H. (2006). The Survivin-Crm1 interaction is essential for chromosomal passenger complex localization and function. EMBO Rep..

[35] Stauber R.H., Rabenhorst U., Rekik A., Engels K., Bier C., Knauer S.K. (2006). Nucleocytoplasmic Shuttling and the Biological Activity of Mouse Survivin are Regulated by an Active Nuclear Export Signal. Traffic.

[36] Trieselmann N., Armstrong S., Rauw J. (2003). Ran modulates spindle assembly by regulating a subset of TPX2 and Kid activities including Aurora A activation. J. Cell Sci..

[37] Vos J.W., Pieuchot L., Evrard J.L., Janski N., Bergdoll M., de Ronde D., Perez L.H., Sardon T., Vernos I., Schmit A.C. (2008). The Plant TPX2 Protein Regulates Prospindle Assembly before Nuclear Envelope Breakdown. Plant Cell.

[38] Brodie K.M., Henderson B.R. (2012). Characterization of BRCA1 Protein Targeting, Dynamics, and Function at the Centrosome: A role for the nuclear export signal, crm1, and aurora a kinase. J. Biol. Chem..

[39] Kaffman A., Rank N.M., O’Neill E.M., Huang L.S., O’Shea E.K. (1998). The receptor Msn5 exports the phosphorylated transcription factor Pho4 out of the nucleus. Nature.

[40] Solsbacher J., Maurer P., Bischoff F., Schlenstedt G. (1998). Cse1p is involved in export of yeast importin α from the nucleus. Mol. Cell. Biol..

[41] Karsenti E., Newport J., Kirschner M. (1984). Respective roles of centrosomes and chromatin in the conversion of microtubule arrays from interphase to metaphase. J. Cell Biol..

[42] Carazo-Salas R.E., Guarguaglini G., Gruss O.J., Segref A., Karsenti E., Mattaj I.W. (1999). Generation of GTP-bound Ran by RCC1 is required for chromatin-induced mitotic spindle formation. Nature.

[43] Kaláb P., Pu R.T., Dasso M. (1999). The Ran GTPase regulates mitotic spindle assembly. Curr. Biol..

[44] Ohba T., Nakamura M., Nishitani H., Nishimoto T. (1999). Self-organization of microtubule asters induced in Xenopus egg extracts by GTP-bound Ran. Science.

[45] Wilde A., Zheng Y. (1999). Stimulation of microtubule aster formation and spindle assembly by the small GTPase Ran. Science.

[46] Kahana J.A., Cleveland D.W. (1999). Beyond Nuclear Transport: Ran-GTP as a Determinant of Spindle Assembly. J. Cell Biol..

[47] Carazo-Salas R.E., Gruss O.J., Mattaj I.W., Karsenti E. (2001). Ran-GTP coordinates regulation of microtubule nucleation and dynamics during mitotic-spindle assembly. Nat. Cell Biol..

[48] Caudron M., Bunt G., Bastiaens P., Karsenti E. (2005). Spatial coordination of spindle assembly by chromosome-mediated signaling gradients. Science.

[49] Kaláb P., Weis K., Heald R. (2002). Visualization of a Ran-GTP gradient in interphase and mitotic Xenopus egg extracts. Science.

[50] Kaláb P., Pralle A., Isacoff E.Y., Heald R., Weis K. (2006). Analysis of a RanGTP-regulated gradient in mitotic somatic cells. Nat. Cell Biol..

[51] Gruss O.J., Carazo-Salas R.E., Schatz C.A., Guarguaglini G., Kast J., Wilm M., Le Bot N., Vernos I., Karsenti E., Mattaj I.W. (2001). Ran induces spindle assembly by reversing the inhibitory effect of importin α on TPX2 activity. Cell.

[52] Gruss O.J., Vernos I. (2004). The mechanism of spindle assembly functions of Ran and its target TPX2. J. Cell Biol..

[53] Nachury M.V., Maresca T.J., Salmon W.C., Waterman-Storer C.M., Heald R., Weis K. (2001). Importin β is a mitotic target of the small GTPase Ran in spindle assembly. Cell.

[54] Wiese C., Wilde A., Moore M., Adam S., Merdes A. (2001). Role of importin-β in coupling Ran to downstream targets in microtubule assembly. Science.

[55] Koffa M.D., Casanova C.M., Santarella R., Köcher T., Wilm M., Mattaj I.W. (2006). HURP is part of a Ran-dependent complex involved in spindle formation. Curr. Biol..

[56] Silljé H.H., Nagel S., Körner R., Nigg E.A. (2006). HURP is a Ran-importin β-regulated protein that stabilizes kinetochore microtubules in the vicinity of chromosomes. Curr. Biol..

[57] Askjaer P., Galy V., Hannak E. (2002). Ran GTPase Cycle and Importins α and β Are Essential for Spindle Formation and Nuclear Envelope Assembly in LivingCaenorhabditis elegans Embryos. Mol. Biol. Cell.

[58] Silverman-Gavrila R.V., Wilde A. (2006). Ran is required before metaphase for spindle assembly and chromosome alignment and after metaphase for chromosome segregation and spindle midbody organization. Mol. Biol. Cell.

[59] Gruss O.J., Wittmann M., Yokoyama H., Pepperkok R., Kufer T., Sillje H., Karsenti E., Mattaj I.W., Vernos I. (2002). Chromosome-induced microtubule assembly mediated by TPX2 is required for spindle formation in HeLa cells. Nat. Cell Biol..

[60] Ribbeck K., Groen A.C., Santarella R., Bohnsack M.T., Raemaekers T., Köcher T., Gentzel M., Görlich D., Wilm M., Carmeliet G. (2006). NuSAP, a Mitotic RanGTP Target That Stabilizes and Cross-links Microtubules. Mol. Biol. Cell.

[61] Sato M., Toda T. (2007). Alp7/TACC is a crucial target in Ran-GTPase-dependent spindle formation in fission yeast. Nature.

[62] Albee A.J., Tao W., Wiese C. (2006). Phosphorylation of Maskin by Aurora-A Is Regulated by RanGTP and Importin beta. J. Biol. Chem..

[63] Meunier S., Vernos I. (2011). K-fibre minus ends are stabilized by a RanGTP-dependent mechanism essential for functional spindle assembly. Nat. Cell Biol..

[64] Maresca T.J., Niederstrasser H., Weis K., Heald R. (2005). Xnf7 Contributes to Spindle Integrity through Its Microtubule-Bundling Activity. Curr. Biol..

[65] Tahara K., Takagi M., Ohsugi M., Sone T., Nishiumi F., Maeshima K., Horiuchi Y., Tokai-Nishizumi N., Imamoto F., Yamamoto T. (2008). Importin-β and the small guanosine triphosphatase Ran mediate chromosome loading of the human chromokinesin Kid. J. Cell Biol..

[66] Ems-McClung S., Zheng Y., Walczak C. (2004). Importin α/β and Ran-GTP regulate XCTK2 microtubule binding through a bipartite nuclear localization signal. Mol. Biol. Cell.

[67] Hagan I.M. (1998). The fission yeast microtubule cytoskeleton. J. Cell Sci..

[68] Peset I., Vernos I. (2008). The TACC proteins: TACC-ling microtubule dynamics and centrosome function. Trends Cell Biol..

[69] Ohkura H., Garcia M.A., Toda T. (2001). Dis1/TOG universal microtubule adaptors-one MAP for all?. J. Cell Sci..

[70] Sato M., Vardy L., Garcia M.A., Koonrugsa N., Toda T. (2004). Interdependency of fission yeast Alp14/TOG and coiled coil protein Alp7 in microtubule localization and bipolar spindle formation. Mol. Biol. Cell.

[71] Al-Bassam J., Kim H., Flor-Parra I., Lal N., Velji H., Chang F. (2012). Fission yeast Alp14 is a dose-dependent plus end-tracking microtubule polymerase. Mol. Biol. Cell.

[72] Brouhard G.J., Stear J.H., Noetzel T.L., Al-Bassam J., Kinoshita K., Harrison S.C., Howard J., Hyman A.A. (2008). XMAP215 Is a Processive Microtubule Polymerase. Cell.

[73] Zheng F., Li T., Jin D., Syrovatkina V. (2014). Csi1p recruits alp7p/TACC to the spindle pole bodies for bipolar spindle formation. Mol. Biol. Cell.

[74] Kakui Y., Sato M., Okada N., Toda T., Yamamoto M. (2013). Microtubules and Alp7-Alp14 (TACC-TOG) reposition chromosomes before meiotic segregation. Nat. Cell Biol..

[75] Sato M., Okada N., Kakui Y., Yamamoto M., Yoshida M., Toda T. (2009). Nucleocytoplasmic transport of Alp7/TACC organizes spatiotemporal microtubule formation in fission yeast. EMBO Rep..

[76] Tang N., Takada H., Hsu K. (2013). The internal loop of fission yeast Ndc80 binds Alp7/TACC-Alp14/TOG and ensures proper chromosome attachment. Mol. Biol. Cell.

[77] Tang N.H., Okada N., Fong C.S., Arai K., Sato M., Toda T. (2014). Targeting Alp7/TACC to the spindle pole body is essential for mitotic spindle assembly in fission yeast. FEBS Lett..

[78] Kudo N., Wolff B., Sekimoto T., Schreiner E.P., Yoneda Y., Yanagida M., Horinouchi S., Yoshida M. (1998). Leptomycin B inhibition of signal-mediated nuclear export by direct binding to CRM1. Exp. Cell Res..

[79] Garcia M., Vardy L., Koonrugsa N. (2001). Fission yeast ch-TOG/XMAP215 homologue Alp14 connects mitotic spindles with the kinetochore and is a component of the Mad2-dependent spindle checkpoint. EMBO J..

[80] Funabiki H., Hagan I., Uzawa S. (1993). Cell cycle-dependent specific positioning and clustering of centromeres and telomeres in fission yeast. J. Cell Biol..

[81] Chikashige Y., Ding D., Funabiki H., Haraguchi T. (1994). Telomere-led premeiotic chromosome movement in fission yeast. Science.

[82] Chikashige Y., Ding D.Q., Imai Y., Yamamoto M., Haraguchi T., Hiraoka Y. (1997). Meiotic nuclear reorganization: Switching the position of centromeres and telomeres in the fission yeast Schizosaccharomyces pombe. EMBO J..

[83] Komeili A., O’Shea E. (1999). Roles of phosphorylation sites in regulating activity of the transcription factor Pho4. Science.

[84] Oshima Y. (1997). The phosphatase system in Saccharomyces cerevisiae. Genes Genet. Syst..

[85] O’Neill E., Kaffman A., Jolly E., O’Shea E. (1996). Regulation of PHO4 nuclear localization by the PHO80-PHO85 cyclin-CDK complex. Science.

[86] Schneider K., Smith R., O’Shea E. (1994). Phosphate-regulated inactivation of the kinase PHO80-PHO85 by the CDK inhibitor PHO81. Science.

[87] Kosugi S., Hasebe M., Tomita M. (2009). Systematic identification of cell cycle-dependent yeast nucleocytoplasmic shuttling proteins by prediction of composite motifs. Proc. Natl. Acad. Sci. USA.

[88] Róna G., Borsos M., Ellis J.J., Mehdi A.M., Christie M., Környei Z., Neubrandt M., Tóth J., Bozóky Z., Buday L. (2014). Dynamics of re-constitution of the human nuclear proteome after cell division is regulated by NLS-adjacent phosphorylation. Cell Cycle.

[89] Marelli M., Aitchison J., Wozniak R. (1998). Specific binding of the karyopherin Kap121p to a subunit of the nuclear pore complex containing Nup53p, Nup59p, and Nup170p. J. Cell Biol..

[90] Makhnevych T., Lusk C.P., Anderson A.M., Aitchison J.D., Wozniak R.W. (2003). Cell cycle regulated transport controlled by alterations in the nuclear pore complex. Cell.

[91] Wu Z., Jiang Q., Clarke P., Zhang C. (2013). Phosphorylation of Crm1 by CDK1-cyclin-B promotes Ran-dependent mitotic spindle assembly. J. Cell Sci..

[92] Hayashi H., Kimura K., Kimura A. (2012). Localized accumulation of tubulin during semi-open mitosis in the Caenorhabditis elegans embryo. Mol. Biol. Cell.

[93] De Souza C.P.C., Osmani A.H., Hashmi S.B., Osmani S.A. (2004). Partial Nuclear Pore Complex Disassembly during Closed Mitosis in Aspergillus nidulans. Curr. Biol..

[94] Arai K., Sato M., Tanaka K., Yamamoto M. (2010). Nuclear compartmentalization is abolished during fission yeast meiosis. Curr. Biol..

[95] Asakawa H., Kojidani T., Mori C., Osakada H., Sato M., Ding D.Q., Hiraoka Y., Haraguchi T. (2010). Virtual Breakdown of the Nuclear Envelope in Fission Yeast Meiosis. Curr. Biol..

[96] Shimoda C. (2004). Forespore membrane assembly in yeast: Coordinating SPBs and membrane trafficking. J. Cell Sci..

[97] Watson M. (1955). The nuclear envelope: Its structure and relation to cytoplasmic membranes. J. Biophys. Biochem. Cytol..

[98] Gonzalez Y., Meerbrey K., Chong J., Torii Y. (2009). Nuclear shape, growth and integrity in the closed mitosis of fission yeast depend on the Ran-GTPase system, the spindle pole body and the endoplasmic reticulum. J. Cell Sci..

[99] Aoki K., Hayashi H., Furuya K., Sato M., Takagi T., Osumi M., Kimura A., Niki H. (2011). Breakage of the nuclear envelope by an extending mitotic nucleus occurs during anaphase in Schizosaccharomyces japonicus. Genes Cells.

[100] O’Day D., Budniak A. (2015). Nucleocytoplasmic protein translocation during mitosis in the social amoebozoan Dictyostelium discoideum. Biol. Rev..

[101] Leo M., Santino D., Tikhonenko I., Magidson V. (2012). Rules of engagement: Centrosome-nuclear connections in a closed mitotic system. Biol. Open.

[102] Nigg E., Blangy A., Lane H. (1996). Dynamic changes in nuclear architecture during mitosis: On the role of protein phosphorylation in spindle assembly and chromosome segregation. Exp. Cell Res..

[103] Andersen S.S. (2000). Spindle assembly and the art of regulating microtubule dynamics by MAPs and Stathmin/Op18. Trends Cell Biol..

[104] Sedbrook J.C. (2004). MAPs in plant cells: Delineating microtubule growth dynamics and organization. Curr. Opin. Plant Biol..

[105] Peterman E.J.G., Scholey J.M. (2009). Mitotic Microtubule Crosslinkers: Insights from Mechanistic Studies. Curr. Biol..

[106] Pellman D., Bagget M., Tu Y., Fink G.R., Tu H. (1995). Two microtubule-associated proteins required for anaphase spindle movement in Saccharomyces cerevisiae. J. Cell Biol..

[107] Loiodice I., Staub J., Setty T.G., Nguyen N.-P.T., Paoletti A., Tran P. (2005). Ase1p organizes antiparallel microtubule arrays during interphase and mitosis in fission yeast. Mol. Biol. Cell.

[108] Yamashita A., Sato M., Fujita A., Yamamoto M., Toda T. (2005). The roles of fission yeast ase1 in mitotic cell division, meiotic nuclear oscillation, and cytokinesis checkpoint signaling. Mol. Biol. Cell.

[109] Fu C., Ward J.J., Loiodice I., Velve-Casquillas G., Nedelec F.J., Tran P.T. (2009). Phospho-Regulated Interaction between Kinesin-6 Klp9p and Microtubule Bundler Ase1p Promotes Spindle Elongation. Dev. Cell.

[110] Garcia M.A., Koonrugsa N., Toda T. (2002). Two Kinesin-like Kin I Family Proteins in Fission Yeast Regulate the Establishment of Metaphase and the Onset of Anaphase A. Curr. Biol..

[111] Chou H.Y., Wang T.H., Lee S.C., Hsu P.H., Tsai M.D., Chang C.L., Jeng Y.M. (2011). Phosphorylation of NuSAP by Cdk1 regulates its interaction with microtubules in mitosis. Cell Cycle.

[112] Shim S., de Castro I., Neumayer G., Wang J. (2015). Phosphorylation of Targeting Protein for Xenopus kinesin-like protein 2 (TPX2) at threonine 72 in spindle assembly. J. Biol. Chem..

